# Understanding of Factors that Enable Health Promoters in Implementing Health-Promoting Schools: A Systematic Review and Narrative Synthesis of Qualitative Evidence

**DOI:** 10.1371/journal.pone.0108284

**Published:** 2014-09-29

**Authors:** Tommy Tsz Man Hung, Vico Chung Lim Chiang, Angela Dawson, Regina Lai Tong Lee

**Affiliations:** 1 School of Nursing, The Hong Kong Polytechnic University, Hung Hom, Kowloon, HKSAR; 2 Health Services and Practice, Faculty of Health, University of Technology, Sydney, Australia; National Cardiovascular Center Hospital, Japan

## Abstract

Health-promoting schools have been regarded as an important initiative in promoting child and adolescent health in school settings using the whole-school approach. Quantitative research has proved its effectiveness in various school-based programmes. However, few qualitative studies have been conducted to investigate the strategies used by health promoters to implement such initiatives. In this study, the researchers conducted a systematic review and narrative synthesis of the qualitative literature to identify important enablers assisting the implementation of health-promoting schools from the perspectives of health promoters. Five enablers have been identified from the review: (a) Following a framework/guideline to implement health-promoting schools; (b) Obtaining committed support and contributions from the school staff, school board management, government authorities, health agencies and other stakeholders; (c) Adopting a multidisciplinary, collaborative approach to implementing HPS; (d) Establishing professional networks and relationships; and (e) Continuing training and education in school health promotion. This highlights the importance of developing school health policies that meet local health needs, and socio-cultural characteristics that can foster mutual understanding between the health and education sectors so as to foster health promotion in children and adolescents.

## Introduction

The concept of Health-Promoting Schools (HPS) evolved in the 1980s and has been regularly advocated as an effective approach to promoting health in schools [1], [2]. The concept of HPS embodies a whole-school approach to community health promotion, in which a broad health education curriculum is supported by the ethos and the environment of the school [3]–[5]. The World Health Organization [WHO] [6] states that “a health-promoting school is one that constantly strengthens its capacity as a healthy setting for living, learning and working”. It is effective in encouraging children to adopt health-enhancing behaviours and in reducing health-compromising behaviours [2], [7]. The HPS approach has been widely accepted by the education sector as an effective and important method of implementing a school health programme [8]–[10]. However, collaboration between the health and education sectors is not always optimal to achieve the common goal of improving students’ health, due their traditionally-rooted role expectations [11], such as the fact that teachers are expected to ensure students’ academic achievement while school nurses aim for behavioural changes among the students [11]. Although the health and education sectors may share the same goal of improving students’ health, their different approaches to the issue and the outcome measures developed are based on different assumptions [11]. Traditionally, educators assume that students are able to make relevant health-related behavioural changes if they have acquired the appropriate knowledge, thus the outcomes are based on cognitive skills such as remembering, understanding, applying, analysing, evaluating and creating [11]. On the other hand, training in the health sector requires healthcare professionals to assess biometric outcomes and the prevalence of diseases, such as body mass index and the prevalence of substance use in the school setting [11]. This fundamental difference between the health and education sectors has led to different understandings of the terms “health education” and “health promotion”, which are often applied differently or interchangeably [12], as well as different theoretical bases used in conducting school-based health interventions [1].

The WHO Ottawa Charter for Health Promotion [13] has inspired healthcare professionals to re-define suitable strategies for health promotion. The Ottawa Charter identified five key action strategies: *building healthy public policy, creating supportive environments, strengthening community action, developing personal skills* and *reorienting health services.* These key strategies have advanced the traditional way of school-based health intervention from focusing on biometrics outcome-based evaluation (which is usually from the health sector perspective) to the multifaceted whole-school approach, which also emphasizes collaboration within and outside schools. However, Croghan, Johnson and Aveyard [14] suggested that historical, political, cultural and contextual sphere influence the health promoters’ potential in practising school health promotion. This means that even in schools that have “explicitly” adopted the HPS initiative, variations occur in the implementation process as well as the outcomes, in addition to the fact that evaluation of the HPS is challenging in itself due to the complexity of school settings and the ambiguity of definitions and understandings of “health education” and “health promotion” by different staff in schools [1], [5], [12]. Therefore, in order to understand how schools successfully implement HPS, process evaluation has been widely suggested by different authors after they have performed intervention studies or systematic reviews [1], [2], [15]. However, they also comment that process evaluation is difficult due to the lack of detailed descriptions of every single step of the health intervention programmes, as well as the fact that it is frequently necessary for implementers to modify the programmes in the complex, ever-changing school settings [1], [2], [15].

As a result, the authors here attempted to use qualitative methods to explore factors that facilitate collaborative action in order to deliver effective HPS from the perspectives of both health and education providers. This review sought to identify:

- The factors that facilitate the delivery of HPS (the enablers), and
- Strategies to better support health promoters in delivering the HPS intervention.

In this paper, we refer to both health and education professionals who contribute to school health education or health promotion as health promoters.

## Methodology

We employed a narrative synthesis methodology in this integrative review of peer-reviewed literature due to the variety of study methodologies, interventions, settings and influencing factors [14]. Narrative synthesis is suitable for synthesizing findings from a range of studies that are insufficiently similar to use specialist synthesis approaches such as statistical meta-analysis and meta-ethnography [16]. A number of systematic reviews have been done that aimed at finding evidence of the effectiveness of the HPS approach [1], [2], [15]. The inclusion criteria in these reviews varied, yet all of the articles included were solely experimental studies. In addition, these reviews discussed and questioned the appropriateness of adopting RCTs to study school health promotion interventions or programmes. For example, the flexible and whole-school approach of HPS, which involves the participation and interaction of the health promoters, makes it too complex to perform a RCT [17]. RCTs involve statistical assumptions that yield large sample sizes, making the interventions expensive and difficult to implement [1], [15]. The heterogeneity of different interventions make the combination of results (e.g. meta-analysis) impossible, and even process evaluations are difficult as the interventions are likely to be implemented poorly [15]. Further, in some cases these intervention- or curriculum-based approaches might not necessarily reflect the whole-school philosophy of HPS [18]. Therefore, incorporating qualitative approaches in research on HPS has been suggested [1], [15], [17], [18], and this systematic review adopts a narrative synthesis approach which targets studies that focus on the whole-school approach of HPS. This review was undertaken according to Popay et al.’s guidance on conducting narrative synthesis in systematic reviews [16].

### Search methods

The search aimed to identify the textual and narrative evidence from the literature on health promoters’ experiences in implementing HPS. Four electronic databases, CINAHL, Ovid, Medline and Web of Knowledge, were searched, as well as The Journal of School Nursing and the Journal of School Health. The databases were searched for the period from 2002 to December 2012, so that the selected articles would be relevant to the current societal and educational climate [19]. The date of the last search was 31 December 2013. A combination of keywords and thesaurus terms was used in the databases: health promoters (or health promotion actors, principals, teachers, school nurses, stakeholders), AND school (or school-based), AND health promotion (or promoting school, comprehensive school health, and coordinated school health), AND qualitative research (or focus groups, grounded theory, semi-structured interviews and framework). The results obtained were then combined with experience, strategy, programme and/or implementation. Reference lists of related articles were reviewed, and experts in the areas were approached to suggest relevant studies. In order to achieve a broad scope of literature, there were no restrictions on language, publication type or study design. Non-English articles were only excluded for eligibility at the full-text assessment stage. Studies were included if (a) the research included health promoters (principals, teachers, school nurses and/or other staff involved in school health promotion), and (b) the study aimed to explore the views of the process of implementing health-promoting schools.

The review employed the PRISMA (Preferred Reporting Items for Systematic Reviews and Meta-Analysis) statement [20]. The PRISMA flow diagram (Figure S1 and Checklist S1) illustrates the process followed in this review.

### Quality appraisal

The Critical Appraisal Skills Programme (CASP) Qualitative Checklist was used to assess the quality of the six included studies [21]. It contains 10 questions (items) which guide researchers in reviewing qualitative studies. Each item was checked “Yes”, “Can’t tell” or “No”. Under each item, there were hints (guiding questions) for consideration. For items that were checked “Can’t tell” or “No” in the appraisal, comments were given independently and discussed between the first and last authors (TTMH & RLTL) in case of disagreement (Table 1). All the six included articles were checked “Yes” for items 1, 2, 3, 7, 8, and 9. The comments are discussed in the following paragraphs.

**Table 1:** Summary of characteristics of the six included articles (part 1/3).

Article	Total sample size	Participants involved	Number of participants contributing qualitative evidence	Study methods	Methods yielding qualitative evidence	Number of interviews/questions, duration, group size	Main results	Quality appraisal CASP Qualitative Checklist (items 1–9 only)
[27]	25	Health promotion specialists, education officers/advisers, client services officers, catering manager, kitchen supervisor, community dietician, school project coordinator, and other school staff	25	Evaluation of the process of a 4-year project within the European Network of Health Promoting Schools in Scotland by multiple case studies	Analysed thematically; in-depth individual interviews; focus group interviews	Forty interviews (baseline and follow-up); duration not mentioned	*Four themes highlighted factors that facilitate translation of HPS principles into practice:*Ownership and empowerment, leadership and management, collaboration, Integration.	1. Yes; 2. Yes; 3. Yes; 4. Can’t tell; 5. Yes; 6. No; 7. Yes; 8. Yes; 9. Yes
[28]	26	Principals and teachers	26	Examining the educational perspectives on implementation of a health-promoting school programme in Sydney	Analysing process drew upon the framework of *complex adaptive systems*; semi-structured individual interviews	Not mentioned	In light of the *complex adaptive systems*: Diversity of component parts and sub-systems of the schools was acknowledged; diversity in and between schools led to diversity of opinion; reaching an agreement on “rules” became difficult; operating context is unique for each school. Failure to recognize the contextual differences led to unrealistic expectations of what schools might achieve; various interaction patterns identified; interactions between schools and the health sector were limited; lots of health-related information which needed prioritization in its flow.	1. Yes; 2. Yes; 3. Yes; 4. Yes; 5. No; 6. No; 7. Yes; 8. Yes; 9. Yes

Inchley et al. [27] did not fully explain how and why they selected the “key stakeholders” for in-depth interviews, or the criteria for student and teacher selection in the focus group interviews (item 4). Pryjmachuk et al. [31] conducted focus group interviews in which all of the participants were female. However, they did not explain why or the potential bias in their results due to the absence of young males (item 4). Keshavarz et al. [28] did not report how they interviewed the staff (item 5). Inchley et al. [27], Keshavarz et al. [28], Morberg et al. [29], and Gugglberger [32] inadequately considered the relationship between researcher and participants in the data collection, discussion and limitations sections of their articles (item 6).

However, all six studies were considered as having passed the quality appraisal after being scrutinized and evaluated by the four authors, as they were important in contributing qualitative evidence relating to health promoters’ experiences in implementing HPS (item 10).

### Data abstraction

All articles were read and the data extracted by two reviewers (TTMH & RLTL) who made decisions regarding inclusion or exclusion. Where possible, consensus was obtained by meeting to compare decisions. In the event of disagreement, a third reviewer (VCLC or AD) read the articles and contributed to decisions and consensus.

### Synthesis

According to [16], one of the purposes of narrative synthesis is to organize findings from included studies in order to identify and list the enablers and the strategies supporting implementation, as well as exploring the relationship between the reported enablers and the supportive strategies. The descriptions may point to a linear process, yet it is a back and forth process in which the synthesis begins as early as in the line-by-line coding [22]. The synthesis was done by data extraction from all of the text labelled as “results” or “findings” in the included studies [22]. Coding was done with the focus on the aim of the present study, at the same time being open-minded to allow for the possibility of different or better fit of codes emerging [22], [23]. Each reviewer did the coding and synthesis independently, and through discussion more abstract or analytical themes began to emerge.

## Results

The study characteristics for the six included articles are summarized in Table 1–3. The full text of articles was included when they met the inclusion criteria and passed the quality appraisal. All other included studies mentioned adopting the WHO HPS. The WHO’s definition of a HPS is ‘one that constantly strengthens its capacity as a healthy setting for living, learning and working’. All included articles are considered to have adopted the whole-school approach, with health promotion supported by the ethos and the environment of the school, rather than just from a narrow perspective reflecting the current health issues of the country or region [2], [11]. Most of the studies were conducted in European countries that possess a long history of implementation of HPS [24], [25]. Three studies drew upon the HPS concept [26]–[28]. Three articles, although not stating explicitly whether they were adopting the HPS concept, were conducted in Sweden [29], [30] and the UK [31], which have joined the HPS-driven SHE Network. All of them were qualitative research studies that identified and reported themes as findings. Two studies [29], [32] used the grounded theory approach in analysing data, but did not aim to generate theory. One study [31] used the framework approach in analysing data. One study [28] presented theory-based qualitative analysis by drawing upon the concept of complex adaptive systems. Five articles have been suggested by experts in the field [14], [19], [33]–[35], but they were excluded due to being in non-school settings, focusing on school nurses’ role only, solely quantitative design, or beyond the time span covered (Figure S1).

**Table 2:** Summary of characteristics of the six included articles (part 2/3).

Article	Total sample size	Participants involved	Number of participants contributing qualitative evidence	Study methods	Methods yielding qualitative evidence	Number of interviews/questions, duration, group size	Main results	Quality appraisal CASP Qualitative Checklist (items 1–9 only)
[28](continued)							Quality and quantity of the information was not always productive; formal and informal feedback loops identified among teachers, students and their parent; formal organization “rules” vs perceived and internalized “rules”; limited sections of curriculum or rules/policies to guide schools in becoming HPS; various attributes of credit and blame to the schools, including award schemes and media coverage; various changes required learning and adaptation, which may not lead to positive change from a health perspective; school behaviour was emergent in nature	
[29]	9	Head school nurses	9	To gain a deeper understanding of how head school nurses in Sweden perceive their leadership in developing school health care	Constructivist grounded theory approach; individual interviews (informal conversation)	Twelve interviews lasting 90 minutes each	*Core category –* Balancing between vague formal goals and strong informal goals. Vague formal goals (subcategory) *:* vague political/state directives for school health care*;* vague organizational delegation for school health care. Strong informal goals (subcategory): Protecting professional boundaries*;* protecting traditional routines. Creating local goals (subcategory): Construction, negotiation and implementation of local goals; meeting school nurses’ expectations of supportive leadership	1. Yes; 2. Yes; 3. Yes; 4. Yes; 5. Yes; 6. Can’t tell; 7. Yes; 8. Yes; 9. Yes

**Table 3:** Summary of characteristics of the six included articles (part 3/3).

Article	Total sample size	Participants involved	Number of participants contributing qualitative evidence	Study methods	Methods yielding qualitative evidence	Number of interviews/questions, duration, group size	Main results	Quality appraisal CASP Qualitative Checklist (items 1–9 only)
[30]	8	School nurses	8	Description of school health nurses’ experiences of the conditions for health promotion work and the aspects that they found important for the success of school health promotion.	Data analysed by content analysis; semi-structured individual interviews	Eight interviews lasting about one hour each	*Three themes that school nurses considered to be important for school health promotion:* Organization, support, and knowledge.	All Yes for item 1–9
[31]	33	School nurses	33	Exploring school nurses’ perspective on managing mental health problems in children and young people.	Data analysed using the framework approach; focus group interviews	Four focus group interviews each involving six to twelve participants and lasting around one hour	*Four main themes on the views of school nurses regarding mental health work in young people:* The mental health of children and young people, organizational issues, barriers to doing mental health work, and facilitators of doing mental health work.	All “Yes” except item 4 (“Can’t tell”)
[32]	23	HP actors	23	Developing a typology of supporting strategies for schools in Austria.	Grounded theory approach; in-depth face-to-face semi-structured interviews; documents	Eighteen interviews lasting between thirty to one hundred and twenty four minutes	*Five types of support for HPS:* Organise exchanges among schools, establish certification and quality control of school health promotion efforts, offer consultation and information concerning health promotion, carry out specific health promotion programmes, and coordinate health promotion actors and information.	All “Yes” except item 6 (“Can’t tell”)

Five key themes were identified in the narrative synthesis that provided insights into the enablers of the delivery of HPS, including supportive strategies to assist health promoters. The findings extracted from the original articles, the examples of codes and the five themes synthesised from the narrative synthesis are outlined in Table 4–9. The themes (enablers) are described below and the synthesised strategies to better support health promoters in implementing HPS are addressed in the discussion.

**Table 4:** Enabler 1 and supportive strategies through narrative synthesis.

Synthesized theme	Examples of codes	Examples identified in original texts (author, year, page)	Possible supportive strategies
Enabler 1:Following aframework/guideline toimplement HPS	Lack of rules/policies	[The] formal rules sometimes were not very simple or were not followed by all agents of the schools. There were not many rules/policies that might guide schools in becoming health-promoting schools” [28].	Simplify rules/policies that guide schools in becoming HPS
	Lack of guidelines	“When I [the school nurse] started five years ago, there was nothing, absolutely nothing. There was no handbook, no common guidelines’ ” [29].	Establish a health-related framework/guideline for school health promoters
	Rules/policies areflexible to beinterpreted; tailored implementation of rules/policies	“We [the school] have to say that we fit into the Department of Education… so our core has to be the parameters that they set. How we interpret [the parameters] has to reflect the needs of the children that we have in our school” [28].	Adopt the framework/guideline in a flexible manner to meet individual schools’ needs
	Huge workload; prioritising work; competing tasks	“Demands usually come that way and it just keeps raining and we [teachers] just can’t keep up with them all, which is why we have to prioritise” [28]. “I [school nurse] think I can set the priorities on my own but I have to stick to the basic programme, which stipulates what I have to do during the school year” [30]. “…Many of the staff said, ‘I haven’t got time to give (to health), apart from the normal sports time’ ” [28].	Prioritise health in school policies
	Time-consuming;HPS as anextracurricularactivity	“One of the difficulties of the health-promoting schools is that it takes away fromsomething else… So if you are going to have one of those, something else has to give a little, because it takes extra time, it takes extra lessons often, to cover the curriculum fully” [28].	Incorporate HPS into the curriculum

**Table 5:** Enabler 2 and supportive strategies through narrative synthesis (part 1/2).

Synthesized theme	Examples of codes	Examples identified in original texts (author, year, page)	Possible supportive strategies
Enabler 2: Obtaining committed support and contributions fromthe school staff, school board management, government authorities, health agenciesand other stakeholders	School management’s support; worked over-time; financial support; government’s help; empowering	“School boards financed additional teachers’ hours with the help of the provincial governments” [32]. “I think [the money] was really important for the school… It gave them a sense of empowerment surrounding their part in the project, that they could spend that money how they felt it should be spent…” [27].	To compensate teachers for extra hours spent on school health promotion
	School management’s support	“One condition of health promotion activities is that there is a [head teacher] and other people in managerial positions who believe that activities to promote health are important” [30].	To obtain the committed support of school management
	Appreciation; media/marketing; politics/politicians; recognising efforts; under-recognising the importance of health	“At my [HP actor] visits to schools I see that they are terribly glad when someone says that they’re doing a great job. That’s very important, also in the media, also from politics, a big recognition” [32]. The ‘Healthy School’ seal of approval is an outwardly visible sign that concepts and measures of school health promotion are applied in a school [32]. [The] certificate was a way to show appreciation for the school. [The health promotion actors] also stressed that: “It’s important to make schools’ achievements visible” [32]. School nurses’ availability was reported as a factor for school security, as well as an advantage in marketing local schools [29].	To recognise health promoters’/schools’ efforts through certification, marketing and political means
	Cultures in society; emphasising academic achievement over children’s health	“[Health] gets pushed to the side. Because then the media comes at you and says why aren’t you teaching kids to read properly and we get the blame. It’s better to let the child get fat than to let the child get low marks. Why? Because that’s what society thinks is more important, a lot of people anyway.” [28].	To cultivate a health-oriented culture in society

**Table 6:** Enabler 2 and supportive strategies through narrative synthesis (part 2/2).

Synthesized theme	Examples of codes	Examples identified in original texts (author, year, page)	Possible supportive strategies
Enabler 2 (continued): Obtaining committed support and contributions from the school staff, school board management, government authorities, health agencies and other stakeholders	Banning the selling of unhealthy food and its advertisement (school management support); choosing of food suppliers and the food provided (food suppliers’ cooperation)	The banning of advertising or selling ‘unhealthy food’ in canteens in many schools had led to changes in the food suppliers’ practices towards providing more healthy options [28].	To ban the sale and advertisement of unhealthy food in schools; to choose appropriate food suppliers that provide healthy food options
	Coordinating; school administration	“It’s the duty of the school administration, it’s a very important function, the task of coordination and to repeatedly suggest what’s new, what you can do, how to develop. Otherwise the schools get lost.” [32].	To coordinate the implementation of health-promoting schools
	Lack of communication between schools and between school and health sectors limited the exchange of knowledge, experiences and resources	“…[The] limitations on interactions between schools and the health sector, and also between the health-promoting schools, there were relatively few exchanges of health-related knowledge, experiences, and resources” [28].	To communicate between schools and between the school and health sectors to facilitate exchanges of knowledge, experiences and resources
	School management’s support; enthusiastic teachers; school management’s motivation; low level of commitment	“It’s scary how crucial [senior management] are because it can be quite frustrating, particularly if you have enthusiastic teachers but they don’t get anything moving forward because there’s no support…” [27]. “It appears that the principal’s individual motivation played a key role in establishing this relationship where it existed” [28].	To assimilate the teachers and the school management’s attitude to school health promotion

**Table 7:** Enabler 3 and supportive strategies through narrative synthesis.

Synthesized theme	Examples of codes	Examples identified in original texts (author, year, page)	Possible supportive strategies
Enabler 3: Adopting a multidisciplinary, collaborative approach to implementing HPS	Organisational linkages; networking; regular meetings	“What I think is missing is to link up the organizations. Cooperation and collaboration across the provinces and with national [health promoters] was rare … Only the social security institutions have a supra-provincial structure and meet regularly” [32].	To organise regular sessions for mutual support, exchanging health-related information and school health promotion experiences between health promoters, between schools, and between schools and parents
	Exchanging information and experiences	“[Schools] exchange experiences and see how the others are doing, with the aim of getting new input” [32].	
	Exchanging information between schools and parents	“A member of the catering staff attended a parents’ evening, providing information about school meals service and the quality of the food [that] they had started to supply” [27].	
	Preserving resources; Working together	[Exchange] encourage[s] reflection in schools, so that schools might be able to save resources by working together on [health promotion] issues, and [so] that they can discuss problems and solutions [32].	
	Exchanging information	Exchang[ing] [information and experiences] can have long-term and sustainable effects that last even if the supporting structure is no longer provided [32].	
	Multidisciplinary; assigning roles/responsibilities collaboration	“If you are going to have a multi-agency, multi-disciplinary group… people do need to have a clear and distinct role within it. And if that’s kind of thrashed out beforehand then I think it makes things easier in terms of the action plan and who’s going to play what part” [27]. “What we are doing now seems to be more of an integral part of the school, but in a much wider sense because [the Catering Manager] and [Deputy Head Teacher] see a lot of each other and we discuss things…” [27].	To adopt a multidisciplinary collaborative approach with clear assignment of roles/responsibilities
	Clear organisational structure; assigning roles/responsibilities; unclear assignment of roles/responsibilities	When there was a clear organisational structure with a clear division of responsibilities, this was good for health promotion activities in school, according to the school health nurses [30]. “I’m [the head school nurse] responsible for the activity manager’s tasks, but again I’m not, I don’t know… they’re not formally delegated to me but I still do the task. Developing healthcare quality, for example, I think the activity manager is also responsible for developing quality” [29].	To establish a clear organisational structure with clear assignment of roles/responsibilities
	Involving students	“The student council has a suggestion box… for new things in the school or something to be improved…” [27].	Incorporate students’ opinions

**Table 8:** Enabler 4 and supportive strategies through narrative synthesis.

Synthesized theme	Examples of codes	Examples identified in original texts (author, year, page)	Possible supportive strategies
Enabler 4: Establishing networks and relationships with stakeholders	Collaborating with specialists	“I’d like to work with a school doctor who’s specialized in children and young people. That would be really good” [30]. “Cooperation between the SHS in my school and the ear specialist in the municipality functions very well, so we send referrals to him and then he comes here and informs us what he and we can do in the school… it’s the same with the skin specialist, she comes here and gives presentations and we discuss pupils’ problems” [30].	To establish professional networks
	Maintaining relationships within and outside schools	[School nurses] described how space and legitimacy for the work of school nurses depended on trust and a good relationship between the head school nurse and the head teachers, as well as local politicians and organisational leaders [29]. Good relationships [between the school nurses and CAMHS] tended to be a facilitator of school nurses… [31].	To maintain a positive relationship with school health promotion stakeholders
	Acting as a coordinator	“[Health promotion specialists play] a crucial role in being “the glue”, in keeping everything together and making contacts” [27].	To assign a full-time coordinator(s) in the implementation of HPS
	Acting as a coordinator; being present	Local negotiations and being a link between school nurses and their decision makers was seen as an important part of the role… Being present in different situations was reported as a priority strategy for visualizing and profiling school health care in the municipalities [29].	
	Interrupted collaboration; working part-time/term-time only	“… you’ve [the school nurse] got six weeks [because you work only during term-time,] then where they [the schools/other supporting agencies] haven’t got that support network or that contact… and I am thinking what am I going to walk into when I walk back in in *[sic]* September” [31].	
	Getting support from peers	“Because we’re all based in one place we’re very lucky… whenever you come back to the office there’ll be somebody there you know and if you’ve had a particular[ly] difficul chat with a young person there’s usually somebody there that you can go back and offload [on]” [31].	To acquire peer support
	Arranging education and training on topics of relationship building and maintenance, and conflict resolution	A good relationship among the school nurses, as well as continuing education… was seen as a priority strategy for strengthening the school nurses’ profession… sometimes in conflict with the head teachers, the head school nurses regularly arranged priority meetings as well as education and training for school nurses in the municipality [29].	To arrange education and training on topics of relationship building and maintenance and conflict resolution

**Table 9:** Enabler 5 and supportive strategies through narrative synthesis.

Synthesized theme	Examples of codes	Examples identified in original texts (author, year, page)	Possible supportive strategies
Enabler 5: continuing training and education in school health promotion	Unclear professional development pathway	“The head school nurses also described that there were no clear goals for their competence development. There were no formalized directives, study programmes or academic degrees for being a head school nurse” [29]. There was a general feeling that there were difficulties and limited opportunities to find suitable courses for developing their role as a head school nurse [29].	To formulate professional developmental plan for school health promoters
	Lack of confidence related to perceived inadequate experiences and knowledge	“…well I [school nurse] said this but I’m not sure whether that was the right thing to say… it’s just having that confidence that you are saying the right thing” [31]. “…I think we’re worried about doing it wrong and we’re worried about doing it badly” [31].	To offer health promoters the opportunities of professional training and education in school health promotion
	Lack of confidence related to perceived inadequate experiences and knowledge (continued)	“You are really inexperienced when you start as a school health nurse, and it takes time before you’ve passed all the training courses. It would have been useful to have more skills related to the SHS included in the basic training” [30]. “I’d like to use the computer more to provide parents, colleagues and politicians with statistics on the well-being of our pupils. I don’t know how to do that, but I’m supposed to be going on a training course” [30].	To offer health promoters opportunities of professional training and education in school health promotion.

### Enabler 1: Following a framework/guideline to implement HPS

Keshavarz et al. [28] and Morberg et al. [29] mentioned that a clear, well-defined and systematic framework of HPS is needed in order to carry out specific programmes successfully. Even policies that were “evidence-informed” might not yield similar outcomes due to the diversity in and between schools, which should be addressed by health promoters and external stakeholders so as to formulate realistic health goals for different schools [28]. Keshavarz et al. [28] also reported that there were not many guidelines that might direct schools to become HPS, nor would the schools follow externally imposed ones. Echoing Keshavarz et al.’s notion of setting up realistic health goals for different schools, Gugglberger suggested that school health promotion should be “precisely structured with certain phases, aims and milestones”, and that “systematic proceedings are necessary for a mutually successful project” ([32], p. 452). Thus, the schools following the HPS framework should consider tailoring their efforts to the individual local context [28]. As reported in the included studies, some health promoters viewed HPS as “add-ons” [27] and “taking extra time” [28], often competing with other programmes or basic routines [30]. As a result, the health promoters, particularly teachers and school nurses as reported, had to prioritise their work, although health promotion was usually given lower priority than “educational achievement” [27], [28], [30].

The staff might lose their enthusiasm for carrying on the school health promotion work, regardless of the apparent immediate results, because a sense of ownership by the individual school was insufficient [27]. In order to develop this sense of ownership, the HPS framework should enable “each member to play a much fuller role in strategic planning and professional decision-making” [27]. The health promoters should also be empowered with the autonomy to set health promotion as the priority [29], with the framework/guideline being flexible in its interpretation, assessment of school needs, developing aims and objectives of school health promotion, allocation of budgets, implementation and evaluation of outcomes [27], [28].

### Enabler 2: Obtaining committed support and contributions from the school staff, school board management, government authorities, health agencies and other stakeholders

A number of studies in this review identified the critical role of the school board management in supporting the implementation of HPS in terms of finance, coordination, policy and commitment [27]–[30], [32]. “[The support] is the duty of the school administration, it’s a very important function… otherwise the school [will] get lost” ([32], p. 452). “It’s scary how crucial [senior management] are because it can be frustrating… because there is no support” ([27], p. 68). The principal’s individual motivation played a key role in establishing relationships and fostering interactions between schools and the health sector, and also between the health-promoting schools [28], [32], which in turn facilitated the exchanges of health-related knowledge, experiences, and resources [28]. As also mentioned in Enabler 1, the sense of ownership and empowerment was related to financial support, especially if the health promoters “could spend that money how they felt it should spent” ([27], p.68). Another request for financial support from the school board management is to pay for the over-time work and extra contributions of the health promoters, particularly the teachers. One of the researchers commented that “[the lack of] financial incentives for teachers was therefore identified as the biggest hindering factor for HP [health promotion] intervention by the actors” [32].

Besides the support from the school board management, the attitudes and the commitment of the school staff played a vital part in the success. A positive attitude such as not seeing the HPS as an “add-on” [27], and the willingness to commit time [28] determined the contribution of the health promoters. For example, huge workloads and time constraints were commonly reported in most of the selected studies [27], [28], [30], [32]. “Many of the staff have said, ‘I haven’t got any time to give [to health], apart from the normal sport time’ ” ([28], p. 1471). Some health promoters were motivated by appreciation for their efforts from external parties (such as the media and politicians), which could sometimes compensate for the lack of incentives [32]. Certificates awarded to the schools which “make the school achievements visible” also served this purpose [32].

While the health promoters perceived such recognition as crucial to their motivation in school health promotion, attitudes towards health or the culture of society also affect health promoters’ motivation: “[health] gets pushed to the side… It’s better that the child be fat than that the child get low marks. *Why?* Because society thinks [academic achievement] is more important, a lot of people anyway” [28, p. 1471, emphasis in original]. This culture might also be transmitted through the media: “Because then the media comes at you and says why aren’t you teaching kids to read properly and we [teachers] get the blame” ([28], p. 1471).

Food suppliers’ commitment contributes to the success of HPS. Keshavarz et al. [28] reported that “the banning of advertising and selling ‘unhealthy food’ in canteens led to the food suppliers’ practice of providing more healthy options” ([28], p. 1471–1472). Therefore the school board management has to make policies to ensure that the food supplied and purchased is healthy for the children [28]. Gugglberger [32] found that the health promoters perceived that they obtained more support when consultation was given to their schools individually. Other support that the stakeholders could offer included health information and manuals, workshops and training in school health promotion, and symposia that facilitated exchange of knowledge and experiences [32].

### Enabler 3: Adopting a multidisciplinary, collaborative approach to implementing HPS

The health promoters believed that a multidisciplinary, collaborative approach brings “long-term and sustainable effects” in school health promotion [27], [32]. It fosters the exchange of experiences and ideas in implementation of HPS [27], [29], [30], [32]. Inchley et al. [27] reported that health promoters like to work in multidisciplinary teams within which experiences and resources can be shared. Some health promoters commented that different people in the team should have a “clear and distinct role” [27] or “a clear division of responsibilities” [30], so that they could focus on their professional areas, such as providing medical care by school nurses [29]–[31], managing catering services by catering managers [27], quality development for health promotion activities by activity managers [29], and administration and management by head school nurses, head teachers and principals [28], [29]. Inchley et al. [27] also found that establishing a common understanding of underlying principles and values and negotiating mutually agreed goals and expectations are crucial in a multidisciplinary team.

Collaboration was not just restricted to adopting a whole-school approach within individual schools [27], [32]. Gugglberger [32] reported that health promoters sought more cooperation and collaboration with other schools in different provinces and countries through various means such as regular meetings, information exchange, mutual sharing and learning, and discussion of problems and solutions. Inchley et al. [27] identified “three spheres” of collaboration: partnership working with external professionals, pupil participation and parental involvement. For example, catering staff were invited to share information about their school meal service at a “parents’ evening”, and a student council gathered student opinions and suggestions that informed changes and improvements in school facilities and policies [27].

### Enabler 4: Establishing professional networks and relationships

Professional networking and relationship building is an essential component in gaining support and consultation for the implementation of HPS [27], [29]–[32]. Health promoters, particularly school nurses, indicated that they liked working with school doctors, paediatric specialists, skin specialists and ear specialists [30]. Some health promoters found it supportive to have a “coordinated school health nurse” [30] or external governmental agencies to help them in school health promotion and coordination, such as the “child and adolescent mental health service provision [CAMHS]” [31] and the “school health service [SHS]” [30]. In case of emergencies, the school nurses had to make appropriate referrals or seek professional advice from private practising specialists [30]. Sometimes the schools invited the specialists to gives presentations and lead discussions on students’ problems [30].

Maintaining good relationships with colleagues within schools as well as with politicians and organizational leaders outside schools has been described as an essential element in the success of HPS [29], [31]. A close working relationship also facilitated communication, information exchange, and sustaining continuous collaborative efforts between the schools and the community, especially when the health promoters responsible for the coordination worked only part-time or during term-time [31]. Further, a “trusting and good relationship” fosters peer supports in the workplace [31]. Inchley et al. noted that a coordinator in school health promotion is “the glue in keeping everything together and making contacts” ([27], p. 69).

### Enabler 5: Continuing training and education in school health promotion

According to the findings of studies included in this review, health promoters seek further training and education to overcome obstacles in implementing HPS projects [29]–[31]. The school nurses described their work as ranging from being lonely at work to having a well-functioning network and support system [30], such as the established professional networks and relationships mentioned in Enabler 4. Health promoters, particularly school nurses, reported a lack of confidence in school health promotion even though they had had training and possessed qualifications in health care [29]. For example, some health promoters asked themselves “how do I integrate this into everyday school life, how should I do this, how does it work?” ([32], p. 451), and a few school nurses expressed feelings of inadequacy in terms of their experience acting as a school health nurse” [30]. Some school nurses worried more about doing counselling or giving out inappropriate health advice to students than about the inaccuracy of their health knowledge [31]. This lack of confidence placed extra stress on health promoters with regard to delivering health promotions in schools [31].

Health promoters, especially school nurses, viewed continuing medical education and training in school health promotion as an important way to deliver quality health promotion in schools [29], [30]. Some school nurses tried to gain more confidence by reading journals and research articles when they perceived a lack of theories and methods in their general health promotion work. However, health promoters might be discouraged from taking such expensive courses, thus financial support is important in this regard ([30], p.160).

## Discussions

This article reports a narrative review of qualitative evidence of the enablers in implementing HPS among health promoters. In this review, qualitative evidence from the included studies demonstrates that commitment from the school administration and management, parents’ and students’ participation, awards and recognition of successful efforts, and collaboration within schools and between schools and the community are important to the implementation of HSP. Communication and health promoters’ competency are also major areas that were found by the studies in this review to determine the success of HPS. Note that due to the heterogeneity of the included studies, the five enablers were generated by synthesising the general concepts from the data presented in the included studies [22]. The themes might overlap each other and the implementation process of HPS is in a dynamic fashion, thus the codes and the data organised in Table 4–9 might also be interpreted differently by different readers.

The issue of the regional guidelines on development of HPS by the World Health Organization in 1996 has attracted more and more health promotion experts from both the education and health sectors to adopt the HPS framework in conducting school-based health intervention programmes and research [1]. During the literature review period in this systematic review, a number of review articles were noted, yet they are not included in the present narrative synthesis, for their data analysis was secondary in nature and they only included quantitative studies such as RCTs [1], [2], [15]. The aims of these reviews include evaluating the effectiveness of school-based promotion interventions [1], [2], identifying methodologies and methodological gaps for evaluating HPS, establishing a conceptual framework for evaluating HPS in South Africa [15], and identifying the effects of student participation in school health promotion [9]. None of them aimed to explore the implementation process of HPS. The role of the school nurse and the concepts of HPS were investigated in a systematic review which included studies done in the 1990s [19] and a content analysis of school staff’s views in Greece in 2008 [35]. All of these articles and reviews, although not included in the current narrative synthesis, are regarded as valuable in providing insightful contents for discussion.

### From policy and guideline establishment to the role delineation of health promoters

Schools possessed different health promotion policies and guidelines, but the interpretations varied among health promoters. Comments on these policies and guidelines ranged from stating that they gave the health promoters more autonomy in implementing HPS to complaining of vague and unclear interpretations that did not help very much in health promotion work and in turn affected the motivation of health promoters. The WHO HPS Framework [36] notes that the users of the guideline should implement it in a way that best fits the individual context and culture; however, as reported in this review, many societies tend to focus on academic achievement [28], which may result in enthusiastic health promoters compromising their motivation and intention in developing the school into a HPS. As evidence in the included studies, adaptation to HPS policies would require a re-prioritization of tasks [28], [30]. The role of fulfilling class teaching by teachers often has to compete with sparing time for classroom health education; this supports the notion of incorporating health education and promotion into the school curriculum [1], [2], [15], [33]. However, this strategy may not necessarily yield positive results, as some health promoters still think that health promotion is not their main role in schools [28], [33]. Interestingly, although the collaborative approach of HPS has been largely emphasized in the included studies, it is seldom mentioned or elaborated how different health promoters see each others’ roles or how they communicate, discuss or clarify among each other. None of the included articles address this possible gap in the perspective of school policy and management, nor could the existing literature provide correlative or comparative studies on this aspect due to the diversity of health promoters’ roles, even among school nurses themselves [19]. For example, Morberg et al. reported that school nurses’ autonomy and legitimacy for work, including school health promotion, actually depended on “trust and a good relationship between the head school nurse and the head teachers” ([29], p. 816), rather than being clearly delineated by existing policies or guidelines. Wainwright, Thomas & Jones [19] also argued that the transition from a teacher-led health promotion conscience to a nurse-led health promotion initiative still raises a lot of conflicts regarding role and professional boundaries. For example, it is not unusual for the existence of school nurses to be based not only on their functional role, but also on the fact that others think that they “bear full responsibility”, and that still others even think that school nurses need not be present permanently in schools [33]. The purpose of the discussion at this point is not to judge whether the existence of certain healthcare professionals, such as school nurses and other specialists, is necessary or suitable in schools, but as a matter of fact that reflects the reality that mutual agreement on the role of different health promoters in implementing HPS has not yet been reached. The authors here argue that the existence of clear policies and guidelines for different health promoters which delineate clearly their roles and responsibilities would improve school nurses’ role and autonomy in school health promotion.

### Coordination is the key to obtaining support and resources

In terms of committed support sought within schools, the top-down managerial approach and the bottom-up commitment approach have been identified from the included articles (Table 4–9) and discussed in the systematic reviews focusing on experimental studies only [1], [2], [9], [15]. Principals and other senior staff who held the manager or supervisor role focused more on strategies for effective HPS implementation, which would draw resources from the community and other stakeholders [17]–[29], [32], such as through marketing and obtaining awards and political recognition?. In contrast to junior teachers or school nurses, they were more focused on personal professional development, such as obtaining professional training and extending their career path [30], [31]. The discussion here is not to compare different health promoters’ position, their knowledge and professionalism or intelligence in any sense, but rather aims to emphasise the diversity and complexity of schools as organisations, in the same way that [28] proposed viewing HPS in light of the complex adaptive system. The authors here argue that in order for a school to obtain all the support required, as identified from the included studies, coordination is the key. While the WHO proposes the HPS as a whole-school approach, the United States Centers for Disease Control and Prevention [CDC] uses the term “Coordinated School Health” [37], which emphasizes the coordinated efforts within the schools and communities. In addition to the suggestion by the health promoters in the included studies to hire full-time school nurses, the CDC stresses hiring a full-time or part-time school health coordinator, who helps to “maintain active school health councils and facilitate health programming in the district and school and between the school and community”, and to facilitate policy and cultural changes in the society [35]. The lack of such coordinated efforts was evidenced in the included studies, in which health promoters frequently lamented the lack of between-school communication, cross-provincial and international exchanges, social recognition and a favourable societal culture, but rarely assimilated the coordination role as their own task. This may be due partially to unsophisticated policies and guidelines, which restricted the potential of health promoters to attain the advanced coordination role, and partially to the current lack of financial support from schools and from the government [1], [2], [15], [35]. However, in a more practical sense, the top-down approach would bring a more effective initiating force to introduce and support this coordination role, while the bottom-up commitment, including the participation of parents and students, is still critical for the sustaining the HPS [2], [9]. The coordination role of health promoters, or the school health promotion coordinators, would thus serve as the medium to bridge the top-down and bottom-up efforts within schools, as well as a resource person who obtains support “horizontally” in the community.

### Limitations

Several limitations should be taken into account when assessing the contributions of the synthesized findings. Not all of the selected studies discussed and evaluated different types of support, even though financial or funding support is considered to be one of the most important factors for the successful implementation of HPS. One study [32] was conducted in a federalist political structure in which, as described by the author, the availability of resources and the financial and personnel autonomy of schools may be influenced under different conditions. Here the narrative synthesis articles have no political standing and therefore political inferences cannot be drawn.

Inchley et al.’s study [27] was drawn from a process evaluation of a 4-year project undertaken within the European Network of HPS in Scotland, although the schools were funded for a 2-year period only. There is no discussion of how the funding facilitated the implementation of HPS, or the effects on HPS after the funding ceased.

A further limitation concerned the range of the population. Included studies were restricted to the UK, Scotland, Sweden, Austria and Australia, and three of them included only school nurses (Table 1–3). This affects the transferability of the findings [38]. Not all of the studies described the details of the interview techniques used or showed the compositions of their participants, which also affected the ‘thickness of data’. The data is ‘thick’ enough if the author includes detailed descriptions and contextual material from which judgements about the trustworthiness of a qualitative research can be made [36], [37]. Therefore, the qualitative evidence presented in this article may be thin and the interpretations and transferability to other contexts may be limited.

Although the authors consider that the articles included here passed the quality appraisal of the CASP tool, their quality varied. Not all of the studies addressed in-depth reflexivity. In addition, the data analysis was not presented in a rigorous way in every study, limiting the interpretations that can be made. Due to the lack of data from these studies, a theory to describe the implementation strategies or process might not be possible at this stage.

## Conclusion

This narrative synthesis review provides qualitative evidence on the enablers and strategies contributing to the successful implementation of HPS. Approaches to implementing HPS may vary among countries and schools owing to the different contexts, thus school health policies that meet local health needs, contexts and cultures need to be formulated. This must involve mutual understanding and relationship building between the health and education sectors in order to promote health to children and adolescents. This article shows that the participation of and support from the school management, collaboration, relationship building and networking between schools and the community are all essential components of the successful implementation of HPS. As found and discussed in this review, health promoters seek a theoretical foundation in order to implement HPS. Further qualitative research is needed to provide a more in-depth understanding of the process of implementation of HPS. A grounded theory approach may be useful in order to develop a substantial theory to describe the process of the successful implementation of HPS.

## Supporting Information

Figure S1 PRISMA flow diagram on different phases of the systematic review.
(PDF)

Checklist S1 PRISMA 2009 Checklist.
(DOCX)
