# Supplementary material for: Understanding of Factors that Enable Health Promoters in Implementing Health-Promoting Schools: A Systematic Review and Narrative Synthesis of Qualitative Evidence
Source: PLoS One. 2014 Sep 29;9(9):e108284. doi: 10.1371/journal.pone.0108284 (PMC4180752; doi:10.1371/journal.pone.0108284)
Supplement: Figure S1 — PRISMA flow diagram on different phases of the systematic review. (PDF) [file pone.0108284.s001.pdf]

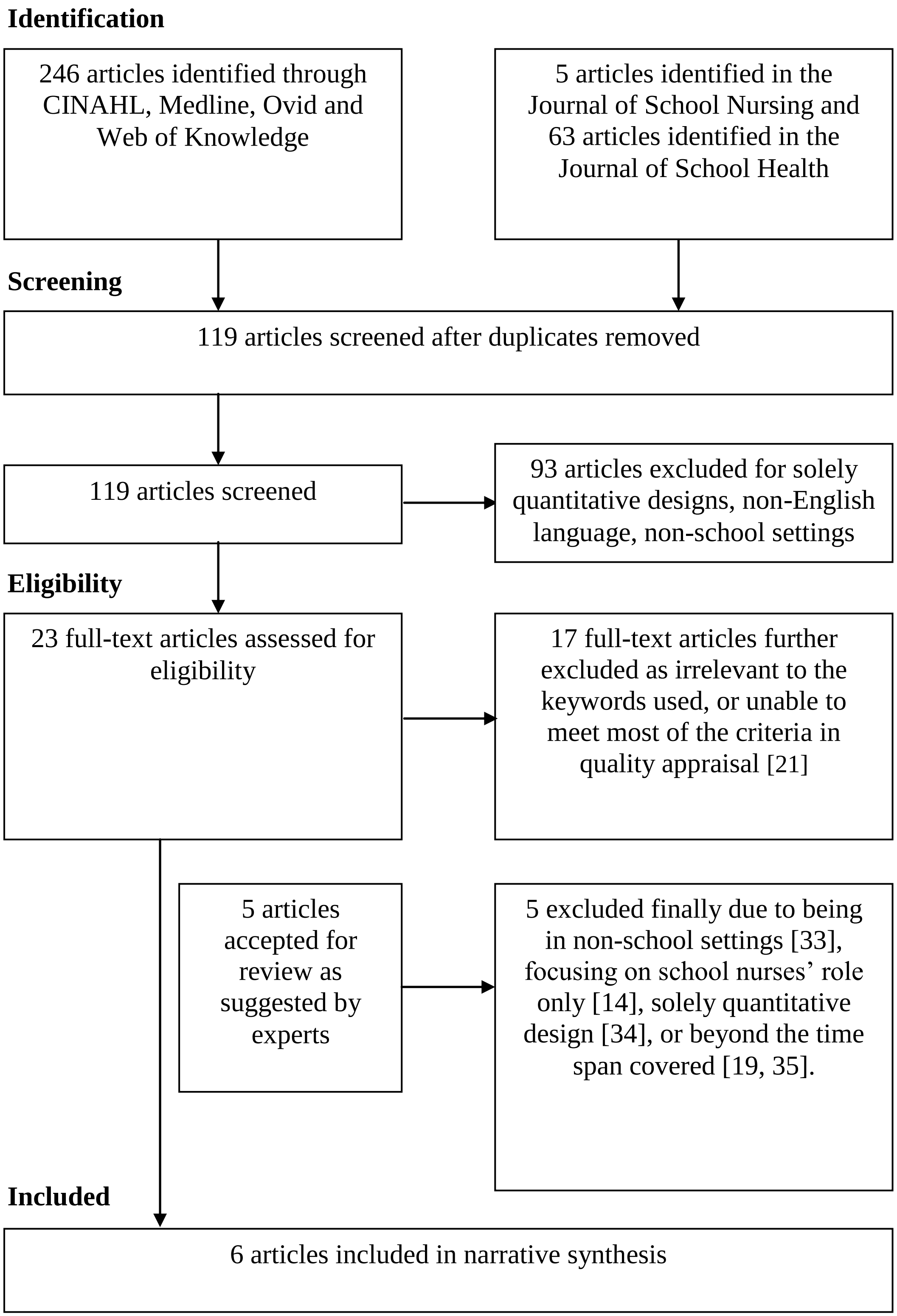

Figure S1. PRISMA flow diagram on different phases of the systematic review
